# Unsupervised anomaly detection of implausible electronic health records: a real-world evaluation in cancer registries

**DOI:** 10.1186/s12874-023-01946-0

**Published:** 2023-05-24

**Authors:** Philipp Röchner, Franz Rothlauf

**Affiliations:** Information Systems and Business Administration, Johannes Gutenberg University, Jakob-Welder-Weg 9, 55128 Mainz, Germany

**Keywords:** Anomaly detection, Outlier detection, Data quality, Quality control, Electronic health records, Medical records, Cancer registration, Neural network, Machine learning, Artificial intelligence

## Abstract

**Background:**

Cancer registries collect patient-specific information about cancer diseases. The collected information is verified and made available to clinical researchers, physicians, and patients. When processing information, cancer registries verify that the patient-specific records they collect are plausible. This means that the collected information about a particular patient makes medical sense.

**Methods:**

Unsupervised machine learning approaches can detect implausible electronic health records without human guidance. Therefore, this article investigates two unsupervised anomaly detection approaches, a pattern-based approach (FindFPOF) and a compression-based approach (autoencoder), to identify implausible electronic health records in cancer registries. Unlike most existing work that analyzes synthetic anomalies, we compare the performance of both approaches and a baseline (random selection of records) on a real-world dataset. The dataset contains 21,104 electronic health records of patients with breast, colorectal, and prostate tumors. Each record consists of 16 categorical variables describing the disease, the patient, and the diagnostic procedure. The samples identified by FindFPOF, the autoencoder, and a random selection—a total of 785 different records—are evaluated in a real-world scenario by medical domain experts.

**Results:**

Both anomaly detection methods are good at detecting implausible electronic health records. First, domain experts identified $8\%$ of 300 randomly selected records as implausible. With FindFPOF and the autoencoder, $28\%$ of the proposed 300 records in each sample were implausible. This corresponds to a precision of $28\%$ for FindFPOF and the autoencoder. Second, for 300 randomly selected records that were labeled by domain experts, the sensitivity of the autoencoder was $22\%$ and the sensitivity of FindFPOF was $26\%$. Both anomaly detection methods had a specificity of $94\%$. Third, FindFPOF and the autoencoder suggested samples with a different distribution of values than the overall dataset. For example, both anomaly detection methods suggested a higher proportion of colorectal records, the tumor localization with the highest percentage of implausible records in a randomly selected sample.

**Conclusions:**

Unsupervised anomaly detection can significantly reduce the manual effort of domain experts to find implausible electronic health records in cancer registries. In our experiments, the manual effort was reduced by a factor of approximately 3.5 compared to evaluating a random sample.

## Background

Cancer registries collect information about cancer patients from various institutions, such as hospitals, medical practices, and pathology laboratories [1]. The information collected is made available to clinical researchers, physicians, and patients to monitor the disease in a given population and to improve the treatment of cancer [1]. To verify the collected information, cancer registries have data quality procedures in place [2]. An essential aspect of data quality is the plausibility of the records that belong to a particular patient: “[D]ata were plausible if they were in agreement with general medical knowledge or information and were therefore feasible” [3]. Plausibility, however, does not necessarily mean that the data accurately describe reality. Causes for implausibility include inadequate and inaccurate information at the time a record is created, as well as human and system errors in the reporting institutions or processing cancer registries. Therefore, cancer registries are interested in identifying implausible reported or processed records [4].

The plausibility of data records can be assessed by rule-based validity checks [3]. Due to the large amount of data in cancer registries, a complete manual data check is usually not feasible. A solution to this problem is the definition of deterministic rules designed by domain experts that identify implausible records [5]. Defining and maintaining deterministic rules for complex error patterns in high-dimensional data is still very labor-intensive. Therefore, statistical and machine learning methods are used to identify quality issues in healthcare [6–8].

Machine learning algorithms can find complex patterns in high-dimensional data. A labeled and representative set of implausible records is, however, usually not available, because implausible records are not necessarily similar to each other, new error patterns can emerge over time, and implausible records are often rare. Therefore, methods to identify implausible records without human guidance, such as unsupervised anomaly detection, are of interest. Anomaly detection methods search for observations that are rare and different from the other observations in a dataset [9]. For example, FindFPOF finds anomalies based on frequently occurring patterns [10, 11]. Newer methods, such as autoencoders, use neural networks to detect anomalies by compressing data [12, 13].

Samples selected by anomaly detection methods can be biased relative to the base distribution. On the one hand, we want to ensure that samples returned by an anomaly detection method have a higher percentage of implausible records than a randomly selected sample. On the other hand, anomaly detection can change the distribution of the variables relative to the base distribution [14]. For example, an anomaly detection method may only suggest records with a certain cancer type or age group. Such a bias can reduce the effectiveness of anomaly detection methods for complete quality control.

This article investigates unsupervised anomaly detection to identify implausible electronic health records in a cancer registry. As a representative example, we study the performance of FindFPOF and an autoencoder approach on a real-world categorical dataset of 21,104 records. The records contain information about the patients, their cancer disease, and diagnostic procedures. Our study considers three different tumor localizations: breast, colorectal, and prostate tumors. As a baseline, we compare the samples selected by FindFPOF and the autoencoder with a randomly selected sample. To verify the plausibility of the records suggested by either the anomaly detection methods or the random selection, medical domain experts manually reviewed the records. We measure the performance of the anomaly detection methods by their precision (percentage of implausible records in the returned samples), sensitivity (percentage of correctly identified implausible records in the random sample), and specificity (percentage of correctly identified plausible records in the random sample). We also examine the differences between the distributions of the samples suggested by FindFPOF, the autoencoder, and the random selection of records.

To the best of our knowledge, the unsupervised detection of implausible electronic health records in cancer registries has not yet been addressed in the literature. Our work contributes to existing work by applying anomaly detection methods to categorical data. So far, low-dimensional numerical datasets have mainly been studied in the healthcare domain [6–8]. In addition, anomaly detection methods have often been evaluated on datasets for classification or with synthetic anomalies [12]. In contrast, this work evaluates anomaly detection methods on a real-world dataset in a real-world scenario, where domain experts manually reviewed 785 different records. Finally, we study the distribution changes created by anomaly detection methods, which are rarely discussed in the literature [14].

The next two sections discuss related work on identifying quality issues in healthcare using anomaly detection and anomaly detection methods for categorical variables. The “Methods” section introduces the dataset studied, the anomaly detection methods, and the evaluation approach. Our experimental results are presented and discussed in the “Results” section. We conclude with remarks on the real-world relevance of our findings and future research directions.

### Anomaly detection for data quality issues in the healthcare domain

Anomaly detection finds various quality issues in healthcare. In [6], anomaly detection identifies malfunctions of data quality rules in clinical decision support systems. For example, data quality rules stop working or alarms are triggered for plausible records due to rule and data changes. In [7], anomaly detection finds safety-related issues in health IT systems. Such issues could be invalid order cancellations or rejections. Estiri et al. identify implausible values in electronic health records with anomaly detection [8].

Existing work compares different anomaly detection methods. In [6], six statistical anomaly detection methods—Poisson Changepoint, Autoregressive Integrated Moving Average, Hierarchical Divisive Changepoint, Bayesian Changepoint, Seasonal Hybrid Extreme Studentized Deviate, and E-divisive with Median models—find malfunctions in four clinical decision support systems. All methods studied are able to identify known malfunctions. Pellet et al. compare accurate-online support vector regression with static and dynamic robust confidence intervals to statistical process control methods [7]. The methods discussed detect anomalies in batch and streaming scenarios. The accurate-online support vector regression with dynamic robust confidence intervals outperforms the traditional methods [7]. In [8], a combination of hierarchical and k-means clustering is compared to statistical detection approaches using standard deviations and Mahalanobis distance. Compared to statistical anomaly detection, the combination of hierarchical and k-means clustering has significantly fewer false positives [8].

The articles discussed evaluate the methods differently. Ray et al. compare the methods studied based on previously labeled data [6]. In [8], the methods are evaluated using lower and upper thresholds for implausible values. The thresholds are generated by literature review or by domain experts and validated using the given data distribution [8]. In [7], there is no ground truth for evaluation. The anomalies of different methods are checked visually [7].

All of the articles mentioned study numerical data. In [6] and [7], the methods are applied to one-dimensional numerical and longitudinal data. In [8], approximately 720 million records with 50 different numerical laboratory results and common health signs are studied. The latter does not take into account the time dependence of the records.

### Anomaly detection methods for categorical variables

In contrast to existing work in healthcare that detects anomalies in numerical data [6–8], cancer registries mainly process categorical data. For categorical data, most of the standard numerical approaches to detect anomalies [15, 16] cannot be used because there is no natural order to the values. The following strategies are available for detecting anomalies in categorical data.

One strategy is to use distance- and density-based anomaly detection methods. Distances for categorical data are often data-dependent [17] and can take into account, for example, the number and frequency of the values. Based on these categorical distances, distance- or density-based anomaly detection methods for numerical data, such as the Local Outlier Factor [18], can be adapted. However, it is difficult to define meaningful distances that capture all relevant relationships between variables and values for diverse and unknown types of anomalies.

Another strategy is to develop methods specifically for categorical data [19], for example, COMPREX and FindFPOF [10, 20]. COMPREX uses several dictionaries to encode data. Each dictionary encodes a partition of the variables in such a way that the total compression cost is minimized. The encoding cost of an observation is its anomaly score [20]. For our experiments, we choose FindFPOF because it is a well-studied classical method for anomaly detection in categorical data [11, 19]. FindFPOF and its parameters are discussed in detail in the “Anomaly detection methods” section.

A third strategy for detecting anomalies in categorical data is to use neural networks, such as deep one-class classification or autoencoders [12, 13]. Neural networks can process categorical variables by using one-hot encoding and appropriate activation and loss functions. Deep one-class classification models optimize the parameters of a neural network to minimize the average distance of the training points to a predefined point in the target space, which is initialized randomly [21]. The anomaly score of an observation is its squared distance to the predefined point in the target space [21]. For our experiments, we choose an autoencoder approach, because it is a widely used deep anomaly detection method that has shown good performance compared to traditional approaches [13, 22]. The autoencoder and its parameters are explained in detail in the “Anomaly detection methods” section.

## Methods

### Dataset

Various medical institutions, such as hospitals, medical practices, and pathology laboratories, collected the records studied during diagnosis. The records were reported electronically to the Cancer Registry Rhineland-Palatinate in Germany between January 2019 and October 2021 [23]. The cancer registry processed the reported information. First, deterministic rules attempted to automatically complete missing values and correct implausible values. Second, the reported records were linked to existing records for the same patient. Third, domain experts manually reviewed and edited records as needed.

**Table 1:** Number of distinct values per variable overall, for only breast, only colorectal, and only prostate tumor records. Each tumor record contains 16 categorical variables describing the disease, the patient, and the diagnostic procedure. The variables are sorted by the decreasing number of distinct values overall

Variable	Explanation	Example	Number of distinct values			
			Overall	Breast	Colorectal	Prostate
ICD-O morphology	Cell type and behavior of tumor	8140/3	127	91	57	33
TNM T	Spread of tumor	4	74	59	33	28
TNM N	Spread of lymph node metastases	3	51	42	25	8
ICD-10 code	Classification of disease	C50.9	30	13	15	2
ICD-O topography	Location of tumor	C50.9	23	9	12	2
TNM M	Presence of remote metastases	1	18	9	15	13
Metastasis	Location of remote metastases	PUL	12	11	11	10
Grading	Amount of abnormality of tumor	2	11	11	10	11
Diagnosis assurance	Diagnostic method	6	7	7	6	7
Lateral localization	Side location of tumor	L	6	5	6	6
c/p-prefix N	Diagnostic method for TNM N	C	5	5	3	3
Diagnosis age (binned)	Age at diagnosis	[59,65)	5	5	5	5
Age at death (binned)	Age at death (if patient has died)	[66,70)	5	5	5	5
Sex	Sex of patient	W	3	3	3	3
c/p-prefix T	Diagnostic method for TNM T	C	3	3	3	3
c/p-prefix M	Diagnostic method for TNM M	C	3	3	3	3

The clinical and population-based Cancer Registry Rhineland-Palatinate collects information on cancer patients living in the German state of Rhineland-Palatinate. In 2021, the federal state of Rhineland-Palatinate had a population of approximately 4.1 million people [24]. In Rhineland-Palatinate, the reporting of cancer cases to cancer registries is mandatory for healthcare providers. Since 1997, population-based information, such as pathological characteristics of cancer cases, has been collected on a legal basis [23]. The collection of clinical information of cancer patients, such as information on local surgery, radiotherapy, and systemic treatments, started in 2016, based on the German Cancer Early Detection and Registry Act of 2013 and its regional implementation of 2015 [23]. Although cancer registries in Germany are mainly organized on a regional level, they have a common structure for data collection [23].

Our dataset consists of 21,104 diagnostic records from patients with breast (ICD-10 codes: C50, D05), colorectal (ICD-10 codes: C18, C19, C20, D01.0, D01.1, D01.2), and prostate (ICD-10 codes: C61, D07.5) tumors. Each record consists of 16 categorical variables. Table 1 shows the different variables, their medical meaning, example values, and the number of distinct values of each variable. The variables ‘diagnosis age’ and ‘age at death’ were binned into five groups such that each bin had the same number of records. After preprocessing, all variables were categorical with 3 to 127 distinct values. We removed duplicates regarding those 16 preprocessed variables from the dataset and filled missing values with a specific value rather than excluding incomplete records or variables from the analysis.

### Anomaly detection methods

FindFPOF and the autoencoder calculate anomaly scores for all records in a dataset without human guidance. Based on these anomaly scores, the records are ranked from normal to anomalous. High ranks correspond to anomalous records and low ranks correspond to normal records. For each method, we selected the most anomalous records (with a high rank according to the anomaly score) for evaluation by the domain experts.

Both methods were trained and evaluated on the entire dataset. Unlike supervised learning, this does not lead to overfitting or a lack of generalization, because the methods are unsupervised and do not learn from labels [15]. If we train FindFPOF and the autoencoder on one dataset and evaluate another dataset with the trained methods, we would expect similar results as in our experiments.

#### FindFPOF

To detect anomalies, the FindFPOF algorithm calculates the Frequent Pattern Outlier Factor (FPOF) [10]. The idea is that normal records have many frequently occurring patterns. Conversely, anomalies have fewer frequently occurring patterns. A pattern is a subset of values such that there is at most one value for each variable. The length of a pattern is equal to the number of its values. A pattern matches a record if the record has all of the values in the pattern. He et al. defined frequent patterns by thresholds for their maximal length and minimal frequency in the dataset [10]. The FPOF of a record is the sum of the frequencies of all matching frequent patterns divided by the number of all frequent patterns. Normal records have a high FPOF and anomalies have a low FPOF [10].

For example, the pattern {Male, Prostate} in Table 2 has a length of two, a frequency of $\frac{1}{3}$, and matches the first record, but it does not match the second and third records. If we consider as frequent patterns all patterns with a maximum length of one and an arbitrary frequency, then there are four different frequent patterns: {Male}, {Female}, {Prostate}, {Breast}. The corresponding frequencies are $\frac{2}{3}$, $\frac{1}{3}$, $\frac{2}{3}$, and $\frac{1}{3}$. Consequently, the FPOF of the first record in Table 2 is $\frac{1}{3}$, of the second and third $\frac{1}{4}$ each. Since a higher FPOF corresponds to more normal cases and men with prostate tumors are more common than the other two records, FindFPOF detects the anomalies as desired.

We set the parameters of FindFPOF as suggested by He et al. and considered frequent patterns as all patterns with a maximal length of five values and a minimum frequency of $10\%$ in the entire dataset [10]. For our dataset, we observed 13,009 frequent patterns.

#### Autoencoder

Autoencoders are neural networks that detect anomalies by their reconstruction error during compression. Autoencoders compress data with an encoder and decompress it with a decoder. To compress data and avoid perfect memorization by the autoencoder, the hidden layers of the autoencoder have fewer neurons than its input and output layers. During training, the parameters of the autoencoder are adjusted to minimize the average reconstruction error. The anomaly score of a record is its reconstruction error. Records with a high reconstruction error are assumed to be anomalies, since anomalies are rare and different [22].

In Table 2, the combination of the sex male and the tumor localization prostate is likely. If perfect memorization is not possible, an encoder could learn to remember the tumor localization and forget the sex of a person during compression; the decoder could learn to reconstruct the sex from the tumor localization. For a man with a prostate tumor, such a decoder would correctly reconstruct the sex as male and the reconstruction error would be low. In contrast, males with breast tumors are unlikely. Thus, a decoder that has learned to reconstruct the sex of a person by the tumor localization would incorrectly reconstruct the sex of a man with a breast tumor as female. The reconstruction error of such a record would be high. Since a higher reconstruction error corresponds to more anomalous records and males with breast tumors are rare, the autoencoder detects the anomalies as desired. This example is only illustrative, since the encoder and decoder of an autoencoder are not directly interpretable.

In our experiments, the autoencoder had three hidden layers with 16, 8, and 16 nodes. The activation functions of the hidden layers were rectified linear units. After each hidden layer, we applied a drop-out layer with a drop-out ratio of 0.2 [25]. We trained the autoencoder using the Adam optimizer with a learning rate of 0.001 and with mini-batches of size 32 for 20 epochs [26]. We used binary cross-entropy as the reconstruction loss.

### Evaluation

#### Implausible records

In our experiments, we define a record as implausible—following Weiskopf et al. [3]—when it contains combinations of values that are not consistent with common medical knowledge or information. Table 2 shows examples of plausible and implausible records. We want to emphasize that even for implausible records, the value of each variable may be valid. Furthermore, the domain experts who reviewed the records could not verify that the data correctly described the patient, the tumor, or the diagnostic procedure [3].

**Table 2:** Three example tumor records with two medical variables, their probability, and their medical plausibility. For the real-world tumor records, domain experts evaluated their medical plausibility. The domain experts could not verify that the information accurately described the person

Medical variables		Probability	Plausibility
Sex	Tumor localization		
Male	Prostate	Probable	Plausible
Female	Prostate	Impossible	Implausible
Male	Breast	Improbable	Plausible

There are several reasons for implausible records. First, records may be implausible because the physicians creating the record have limited or inaccurate information about the tumor or the patient. Second, there may be problems with data collection or processing. These problems may be caused by systems or people in the reporting institutions and the receiving cancer registry. Third, the rules for coding tumors, such as ICD-O codes, may change over time. Finally, there may be differences between the diagnoses of different physicians, as well as between the reporting physicians and the domain experts processing the records [27].

**Fig. 1:**
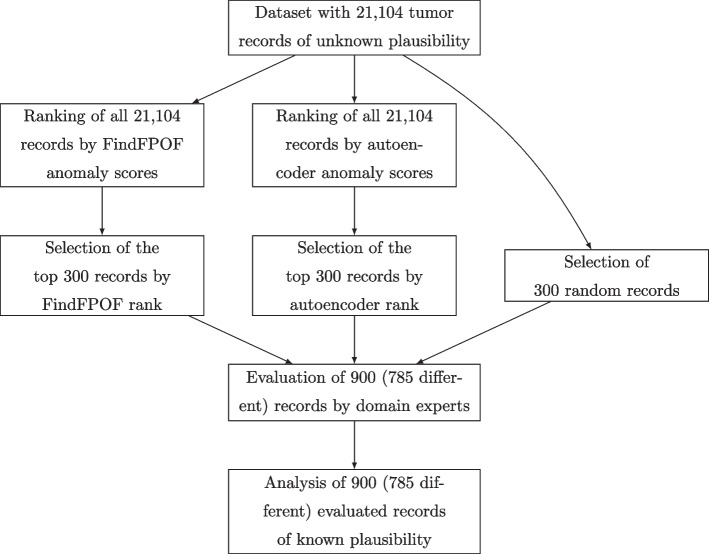
Selection and evaluation approach of tumor records. We separately rank all 21,104 records with unknown plausibility by the FindFPOF and autoencoder anomaly scores. From the 21,104 ranked records, we select the 300 highest-ranked (most anomalous) records according to either FindFPOF or the autoencoder. Additionally, we randomly select 300 records as a baseline. Out of a total of 900 selected records, 785 were different. Domain experts evaluated the plausibility of these 785 different records. The 900 (785 different) evaluated records with known plausibility were analyzed for this study

#### Selection and evaluation approach of tumor records

Figure 1 shows the selection and evaluation approach of tumor records in our experiments. All 21,104 records were ranked separately by the FindFPOF and autoencoder anomaly scores. Domain experts then assessed the plausibility of the records from three different samples. First, the domain experts analyzed the 300 most anomalous (highest-ranked) records returned by either FindFPOF or the autoencoder. Second, we randomly selected 300 from the entire 21,104 records. This random selection is the baseline for evaluating the performance of the anomaly detection methods and is used to estimate the probability of a record to be anomalous.

To obtain an accurate estimate of the probability that a record is implausible, the size of the random sample should be large enough. Confidence intervals for this probability can be calculated based on the total number of records in the random sample and the number of implausible records in the random sample using a two-tailed exact binomial test. When designing the experiments and selecting the size of the random sample, we did not know how many records in the random sample would be implausible. Therefore, we calculated the maximum width of the confidence intervals for a random sample of 300 records and an arbitrary number of implausible records. When evaluating 300 random records, the $95\%$ confidence interval that a random record is implausible is contained in an interval that has at most a with of 0.12. This means, that for a random sample of 300 records, where *k* records are implausible, the probability that a random record is implausible is in the interval $\left[ \frac{k}{300}-0.06, \frac{k}{300}+0.06\right]$ with a probability of 0.95. For a known number of implausible records *k*, the width of the $95\%$ confidence interval may be smaller. Since the confidence interval is sufficiently small, we choose 300 records to evaluate each method.

The 900 records selected contained 785 different records. Manual evaluation of the 785 records is feasible, but time-consuming for domain experts. Since the domain experts are specialized in tumor localization, one out of seven domain experts evaluated each record according to the tumor localization of that record. For each record, the domain experts decided whether it was plausible or implausible. Records that were selected by more than one method were evaluated only once. The experts did not know which method suggested a record.

#### Evaluation metrics

Based on the manual assessment of the domain experts, we calculated the precision of each method as the percentage of implausible records per selected sample. Because we do not know all implausible records in the entire dataset, metrics that require labeling the entire dataset, such as sensitivity and specificity, could not be calculated for all records.

We calculate the sensitivity and specificity of the anomaly detection methods on the random sample that was labeled by the domain experts. The FindFPOF and autoencoder rankings of the 300 randomly selected records were transformed into binary labels by selecting *k* records as anomalies. The *k* highest-ranked records are labeled as anomalies, and the remaining $300-k$ lowest-ranked records are labeled as normal. Based on these binary labels and the domain experts’ evaluation, we calculated the sensitivity and specificity of the anomaly detection methods on the random sample.

## Results

### Precision on the individual samples per tumor localization

We examine the number of implausible records in the three different samples and the precision of the corresponding methods. Table 3 shows the number of implausible records $\#impl$ in each sample overall (all) and for each tumor localization (breast, colorectal, or prostate). We also show the precision $\frac{\#impl}{n}$, calculated as the number of implausible records $\#impl$ over the number of records *n* for that sample and tumor localization.

**Table 3:** Number of selected and implausible records overall and per tumor localization in each sample. FindFPOF and the autoencoder had a higher precision overall and for each tumor localization than the baseline. In the random sample, $8\%$ of all records and $2\%$ of the breast records were implausible. For the autoencoder sample, $28\%$ of all records and $10\%$ of the breast records were implausible. FindFPOF and the autoencoder selected more records from those localizations that had a higher percentage of implausible records in the random sample. For the random sample, $18\%$ of the colorectal records were implausible, while only $2\%$ of the breast records were implausible. Thus, the autoencoder and FindFPOF returned more colorectal records (approximately two-thirds of the records are colorectal) that had a higher percentage of implausible records (approximately one-third). In contrast, the samples returned by the autoencoder and FindFPOF contain a lower percentage of breast records ($28\%$ and $14\%$, respectively) than both the random sample and the full dataset ($54\%$ and $58\%$, respectively). For each sample, the tumor localizations with the highest number of selected and implausible records are highlighted

	Records		Tumor localization			
			All	Breast	Colorectal	Prostate
Full dataset	All	*n* $\left( \frac{n}{n_{all}} \right)$	21,104 (100%)	**11,573 (54%)**	6995 (34%)	2536 (12%)
Random sample	Selected	*n* $\left( \frac{n}{n_{all}} \right)$	300 (100%)	**172 (58%)**	87 (28%)	41 (14%)
	Implausible	$\#impl$ $\left( \text{ precision: } \frac{\#impl}{n}\right)$	23 (8%)	4 (2%)	**16 (18%)**	3 (8%)
Autoencoder sample	Selected	*n* $\left( \frac{n}{n_{all}}\right)$	300 (100%)	85 (28%)	**193 (64%)**	22 (8%)
	Implausible	$\#impl$ $\left( \text{ precision: } \frac{\#impl}{n} \right)$	83 (28%)	9 (10%)	**67 (34%)**	7 (32%)
FindFPOF sample	Selected	*n* $\left( \frac{n}{n_{all}}\right)$	300 (100%)	40 (14%)	**200 (66%)**	60 (20%)
	Implausible	$\#impl$ $\left( \text{ precision: } \frac{\#impl}{n} \right)$	83 (28%)	3 (8%)	**65 (32%)**	15 (24%)
All samples	Selected	Total (different)	900 (785)	297 (266)	480 (406)	123 (113)
	Implausible	Total (different)	189 (157)	16 (14)	148 (124)	25 (19)

For all tumor localizations, $28\%$ of the FindFPOF and autoencoder samples were implausible. This number is approximately a factor of 3.5 higher than the $8\%$ of implausible records found in the random sample. We observe the same behavior for each of the three tumor localizations. The three tumor localizations studied are medically quite different and had percentages of implausible records between $2\%$ and $18\%$ in the random sample. For each tumor localization, the FindFPOF and autoencoder sample contained more implausible records than the random sample. For example, $18\%$ of the randomly selected colorectal records, $34\%$ of the colorectal records identified by the autoencoder, and $32\%$ of the FindFPOF colorectal records were implausible.

Thus, both anomaly detection methods can detect implausible records with medically and qualitatively significantly different tumor localizations. Nevertheless, the majority of the suggested anomalies were plausible.

### Precision on the individual samples over number of selected highest-ranked anomalies

**Fig. 2:**
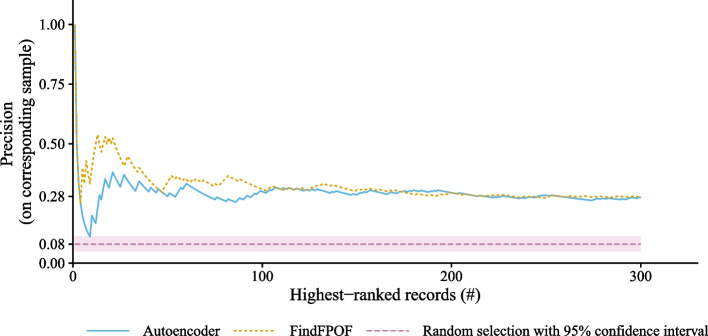
Precision $\frac{\#impl}{n}$ over the number *n* of selected highest-ranked (most anomalous) records in the entire dataset. We selected the *n* most anomalous records of the entire dataset according to the anomaly score of each method. For less than 100 selected records, FindFPOF finds more implausible records than the autoencoder. For more than 100 selected records, the performance of FindFPOF and the autoencoder is approximately equal

Are high-ranked records more likely to be implausible than low-ranked records? In general, we selected the 300 records of all 21,104 records with the highest rank according to the anomaly score returned by the autoencoder or FindFPOF. We now reduce the number of selected records *n* and consider fewer highest-ranked anomalies. We investigate whether a reduction of the number of selected records *n* changes the precision of the investigated methods. Therefore, for each method, Fig. 2 plots the precision $\frac{\#impl}{n}$ calculated as the number of implausibilities $\#impl$ over the number of records considered *n*. We show results for less than 300 records. In addition, Fig. 2 shows the $95\%$ confidence interval of the precision of the random selection to be $8\%$, based on a two-tailed exact binomial test, when 23 of 300 randomly selected records are implausible.

The results show that FindFPOF outperforms the autoencoder approach when we select fewer than 100 records. For example, the 80 highest-ranked (most anomalous) records according to FindFPOF contain 28 ($35\%$ of the sample of 80 records) implausible records. In contrast, the 80 records with the highest autoencoder rank (most anomalous) contain only 21 ($26\%$ of the sample of 80 records) implausible records. When more than the approximately 100 highest-ranked (most anomalous) records are selected, the precision drops slightly to a similar level. As expected, the precision of FindFPOF and the autoencoder is always higher than in the random sample.

### Sensitivity and specificity on the random sample

**Fig. 3:**
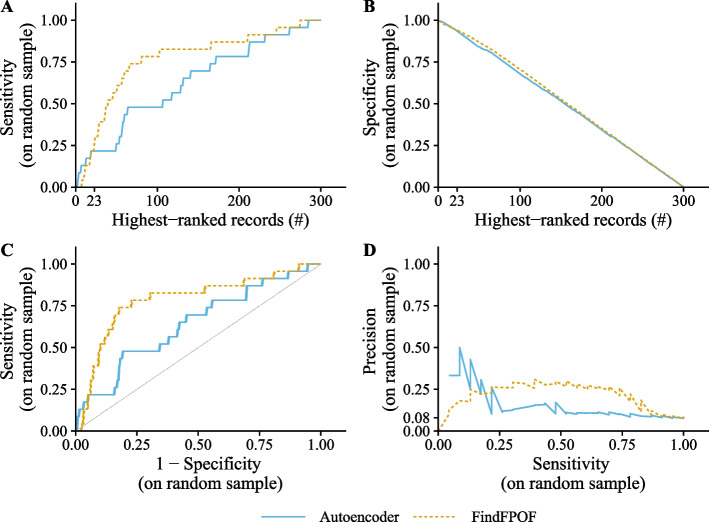
Performance of both anomaly detection methods on the labeled random sample. The top row shows the sensitivity (**A**) and specificity (**B**) over the number of selected highest-ranked (most anomalous) records in the random sample. We selected the most anomalous records in the random sample according to the anomaly score of each method. The bottom row shows the ROC curve (**C**) and the precision-sensitivity curve (**D**). For more than approximately 25 selected records, FindFPOF has a higher sensitivity than the autoencoder. The specificity of both methods decreases similarly

We calculate the sensitivity and specificity of the anomaly detection methods studied on the completely labeled random sample of 300 records. The binary labeling of the randomly selected 300 records based on the anomaly ranking is described in the “Evaluation metrics” section. The top row of Fig. 3 shows the sensitivity (A) and specificity (B) of FindFPOF and the autoencoder on the random sample depending on the number of records in the random sample selected as anomalies. The bottom row shows the corresponding ROC curve (C) and the precision-sensitivity curve (D).

In the random sample, 23 out of 300 records are implausible. When the 23 highest-ranked (most anomalous) records of the random sample are selected according to each anomaly detection method, the autoencoder correctly detects 5 and FindFPOF 6 of the 23 implausible records as implausible (true positives). Similarly, the autoencoder correctly identifies 259 and FindFPOF 260 of the 277 plausible records as plausible (true negatives) when 23 of the 300 records are selected as anomalies by each method. Consequently, the sensitivity of the autoencoder is $22\%$ and of FindFPOF 26% on the random sample for 23 selected anomalies. The specificity of both anomaly detection methods is $94\%$. When more than 23 of the highest-ranked (most anomalous) records are selected, the sensitivity of FindFPOF is higher than or equal to the sensitivity of the autoencoder. In contrast, the specificity of both methods decreases similarly. Overall, FindFPOF detects the implausible records of the random sample better than the autoencoder.

### Overlap between samples for selected and implausible records

Some records were selected by more than one method. Therefore, we analyze the overlap in the samples returned by FindFPOF and the autoencoder separately for selected records and implausible records.

**Fig. 4:**
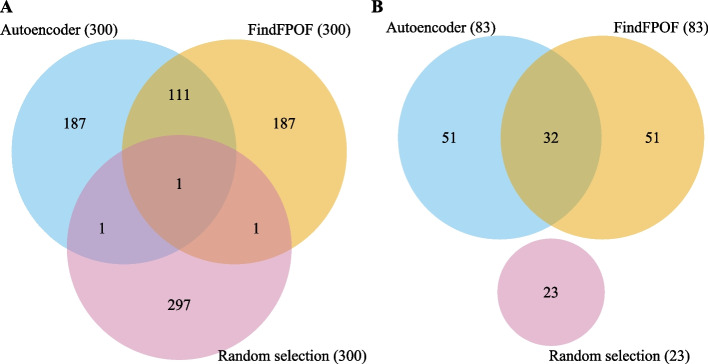
Number of records in one or more samples. The left part (**A**) shows the number of records selected by each method; the right part (**B**) shows the number of implausible records. The FindFPOF and the autoencoder samples had 112 records in common ($37\%$ of the 300 records per sample). Of these 112 jointly detected records, 32 records or $29\%$ are implausible

The last two rows of Table 3 show the total number and the number of different selected records and implausible records for all three samples. The left part (A) of Fig. 4 plots the number of records contained in one or more samples. The right part (B) of Fig. 4 shows the number of implausible records that were selected by FindFPOF, the autoencoder, or at random.

First, we discuss the number of records returned jointly by FindFPOF and the autoencoder. In total, 114 of the 785 different records were contained in multiple samples. The autoencoder and FindFPOF samples had 112 records in common ($37\%$ of the 300 records per sample).

Second, we discuss the number of implausible records returned jointly by FindFPOF and the autoencoder. The domain experts evaluated 157 of the 785 different selected records as implausible. Of these 157 different implausible records, 32 were included in both the FindFPOF and the autoencoder samples. Thus, $29\%$ of the records returned jointly by FindFPOF and the autoencoder were implausible (32 implausible of 112 jointly selected records). Overall, the overlap between the FindFPOF and autoencoder samples is high.

### Percentage of selected anomalies per tumor localization

Does the number of records selected by FindFPOF and the autoencoder differ by tumor localization? For each tumor localization (all, breast, colorectal, and prostate), Table 3 shows the number of records *n* for the full dataset, as well as the random, autoencoder, and FindFPOF samples. The table also shows the percentage of selected records $\frac{n}{n_{all}}$ per sample and tumor localization calculated as the number of records with a particular tumor localization in a sample *n* over the total sample size $n_{all}$. The number $\#impl$ and the percentage $\frac{\#impl}{n}$ of implausible records are described in the “Precision on the individual samples per tumor localization” section.

For the full dataset, the majority of records ($54\%$ of 21,104 records) describe breast tumors. In contrast, only $12\%$ of the total dataset are prostate tumor records. For anomaly detection methods, the distribution of the returned samples is different. For example, the 300 records returned by the autoencoder contain only $28\%$ breast records, which is less than the $54\%$ in the entire dataset. Conversely, the autoencoder sample has more colorectal records, $64\%$ of 300 records compared to only $34\%$ of 21,104 records overall. Thus, breast records are underrepresented and colorectal records are overrepresented in the sample returned by both FindFPOF and the autoencoder. Similarly, prostate records are overrepresented in the FindFPOF sample and slightly underrepresented in the autoencoder sample.

Although $54\%$ of the records in the entire dataset describe breast tumors, only $2\%$ of the randomly selected breast records are implausible. In contrast, the random sample contains $28\%$ of colorectal records, of which $18\%$ of 87 records are implausible. The prostate records are in between. We expect that a well-functioning anomaly detection method would focus on tumor localizations with a higher percentage of implausibilities. In fact, both anomaly detection methods select more colorectal records with a higher percentage of implausible records than breast records. For FindFPOF and the autoencoder, approximately two-thirds ($66\%$ and $64\%$, respectively) of the 300 records selected in each sample are colorectal tumor records. In contrast, the percentage of breast records in the FindFPOF and autoencoder sample ($14\%$ and $28\%$ of each sample with 300 records) was lower than in a random sample. As a result, the autoencoder and FindFPOF favor colorectal tumors because a randomly selected colorectal record has a higher chance of being implausible.

### Diversity of implausible records per selected sample

We study the diversity of the implausible records in the FindFPOF, autoencoder, and random samples. For each sample of implausible records, we analyze the number of distinct values per variable. For example, the number of distinct values for the variable sex in the entire dataset is three: female, male, and diverse. Table 4 shows the number of distinct values per variable of the implausible records in the autoencoder (AE), FindFPOF (FF), and random (RS) samples for all tumor localizations studied (overall) and breast, colorectal, and prostate tumors separately. In each of these samples with implausible records, the number of distinct values per variable can be at most as high as the number of distinct values in the entire dataset (see Table 1).

**Table 4:** Number of distinct values per variable for each sample with implausible records. The number of distinct values per variable is shown for the autoencoder (AE), FindFPOF (FF), and the random selection (RS), as well as for all tumor localizations studied (overall), and only for breast, colorectal, and prostate tumors. The implausible records identified by the autoencoder are more diverse than those identified by FindFPOF, overall, for breast tumors, and colorectal tumors. For prostate, the implausible records of the FindFPOF sample are more diverse. The randomly selected implausible records are much more homogeneous than the implausible records of the other two samples. We highlight the highest number of distinct values. The variables are sorted by the decreasing number of distinct values in the complete random sample

Variable	Number of distinct values											
	Overall			Breast			Colorectal			Prostate		
	AE	FF	RS	AE	FF	RS	AE	FF	RS	AE	FF	RS
ICD-10 code	**21**	15	11	**6**	1	2	**13**	12	8	2	2	1
TNM T	**12**	9	10	**6**	0	2	**10**	6	6	6	6	3
ICD-O topography	**17**	13	9	**5**	1	2	11	11	6	1	1	1
Grading	**9**	7	7	**6**	2	2	**8**	7	6	4	4	2
ICD-O morphology	**23**	19	6	**8**	2	2	17	17	4	4	**5**	1
TNM N	**9**	5	5	**4**	0	2	**8**	4	5	4	4	2
Diagnosis age (binned)	5	5	5	3	3	3	5	5	4	2	**4**	2
Age at death (binned)	5	5	5	2	2	1	5	5	4	2	**4**	1
Lateral localization	**5**	4	4	**3**	1	3	**5**	4	4	3	3	1
TNM M	**10**	7	4	**2**	1	2	**7**	5	3	5	5	2
Metastasis	7	7	4	**4**	3	2	5	5	2	2	**4**	2
Diagnosis assurance	**7**	6	3	**4**	2	2	5	5	2	4	4	1
c/p-prefix T	3	3	3	2	0	2	3	3	3	2	2	2
c/p-prefix N	3	3	3	2	0	2	3	3	3	2	2	2
c/p-prefix M	3	2	3	**2**	1	1	3	2	3	2	2	2
Sex	2	2	2	1	1	1	2	2	2	1	1	1

Overall, for breast and colorectal tumors, neither FindFPOF nor the random sample had more distinct values in any variable than the implausible records of the autoencoder sample. For the implausible records of all three tumor localizations studied, in 9 of 16 variables the implausible records of the autoencoder sample had more distinct values than the records of the FindFPOF and the random samples. For breast records, in 11 of 16 variables, and for colorectal records, in 6 of 16 variables, the implausible records of the autoencoder sample had more distinct values than the implausible records of the other two samples. Only for prostate records did the implausible records identified by FindFPOF have more distinct values in 4 of 16 variables than in the autoencoder and random samples.

In summary, the implausible records identified by the autoencoder are more diverse overall, as well as for breast and colorectal tumors, than those identified by FindFPOF. For prostate, the implausible records of the FindFPOF sample are more diverse. As expected, the randomly selected implausible records are much more homogeneous than the implausible records of the other two samples, since the random sample contains much fewer implausible records.

### Anomaly ranking of all identified implausible records

**Fig. 5:**
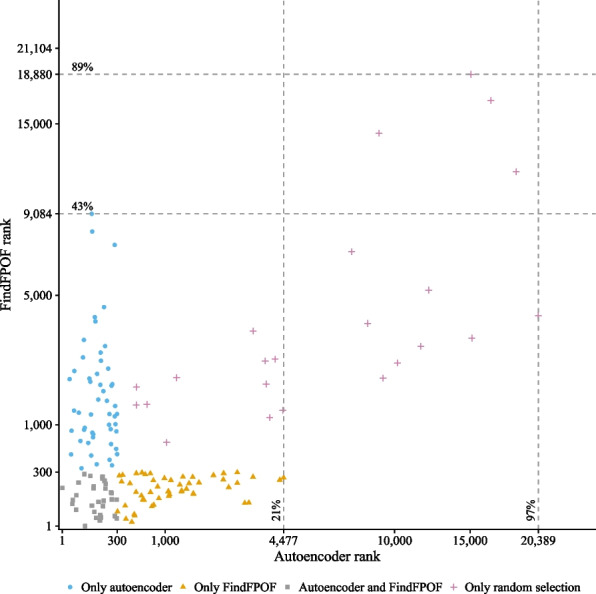
FindFPOF anomaly ranking over autoencoder anomaly ranking for the 157 different implausible records in the FindFPOF, autoencoder, and random samples. Each implausible record is ranked based on the FindFPOF anomaly score and the autoencoder anomaly score. High ranks correspond to anomalous records and low ranks correspond to normal records. The autoencoder finds the implausibilities identified by FindFPOF earlier than the other way around. All implausible records of the FindFPOF sample are in the 4477 ($21\%$ of all 21,104 records) highest-ranked records of the autoencoder. The implausible records of the autoencoder sample are in the 9084 ($43\%$ of all 21,104 records) highest-ranked records of FindFPOF. Note that the scales are square root transformed

We examine how each anomaly detection method evaluated the implausible records of the random sample and the other anomaly detection method. Therefore, we compare the FindFPOF and autoencoder anomaly rank of each found implausible record. For each of the 157 different implausible records found, Fig. 5 shows the FindFPOF anomaly rank over the corresponding autoencoder anomaly rank. The scales are square root transformed. As shown in the right part (B) of Fig. 4, we have four groups of implausible records: implausible records detected only by the autoencoder (●, 51 records), implausible records detected only by FindFPOF (▲, 51 records), implausible records that are in both the FindFPOF and autoencoder samples (■, 32 records), and implausible records that are only in the random sample (✚, 23 records). For each of the implausible records that are only in the autoencoder or random sample, we highlight the lowest FindFPOF rank with horizontal dashed lines. Similarly, we highlight the lowest autoencoder rank of all implausible records that are only in the FindFPOF or random sample with vertical dashed lines. The annotation of each dashed line is the corresponding lowest rank and its percentage of the total 21,104 records.

If we sort all records according to their FindFPOF anomaly score, we would have to evaluate 9084 records, $43\%$ of all 21,104 records, to find all 51 implausible records that are returned only by the autoencoder. Conversely, to find all 51 implausible records that are only in the FindFPOF sample with the autoencoder, we would have to evaluate 4477 records, $21\%$ of all 21,104 records. Thus, the autoencoder identifies the implausible records that are only returned by FindFPOF earlier than the other way around.

To allow the autoencoder to find all 23 implausible records that are only in the random sample, it is necessary to evaluate 20,389 records ($97\%$ of all 21,104 records). For FindFPOF, one needs to evaluate 18,880 records ($89\%$ of all 21,104 records). So, as expected, some implausible records in the whole dataset are not detected well by the autoencoder and FindFPOF.

## Discussion

Unsupervised anomaly detection methods could be an essential part of data quality control in cancer registries. Whenever records are selected for quality control without explicitly defining error patterns, anomaly detection could select these records. These anomalous records would then be reviewed by domain experts. As our experiments show, this could reduce the manual verification effort of domain experts by a factor of approximately 3.5 compared to randomly selecting records (the percentage of implausible records is increased from $8\%$ in the random sample to $28\%$ in the anomalous samples of 300 records each). Our results also show that the precision of each method could be further increased by checking fewer highest-ranked (most anomalous) records, for example, 100 instead of 300 records. Finally, the anomaly detection methods found more diverse implausibilities compared to the random selection. This is important because more diverse implausibilities can point to many different root causes of quality issues. Based on the implausibilities found this way, cancer registries can improve existing processes, implement new rule-based validity checks, and train users. In other words, anomaly detection methods can detect more unknown and diverse implausibilities than traditional quality control approaches.

The anomaly detection methods discussed could also find implausible records for different tumor localizations. In our experiments, the breast, colorectal, and prostate tumors studied were medically and qualitatively quite different (between $2\%$ and $18\%$ of 300 randomly selected records are implausible). For all tumor localizations investigated, the methods studied detected implausible records well. Moreover, the records of most tumor localizations share the 16 variables studied above. Thus, we could apply anomaly detection methods to records of other tumor localizations and would expect similar results.

Implausibilities also occur in records of other medical events. So far, we have only examined tumor records created at the time of diagnosis. Clinical cancer registries, however, also collect information about other medical events, such as surgery, radiotherapy, systemic therapy, and side effects. For each of these medical events, anomaly detection methods could find implausibilities. Among the records describing medical events in clinical cancer registries, the diagnosis records studied are one of the most complex in terms of the number of variables and values. Therefore, we would expect a similar performance when applying the suggested anomaly detection methods to other types of records. Until now, we have considered different records of the same person independently. Implausibilities between different medical events of the same person could be detected by anomaly detection methods for discrete event sequences [28].

Anomaly detection methods can detect anomalies in high-dimensional data. The data of other tumor localizations or medical events may have more dimensions than the data we studied in our experiments. In high-dimensional data, some anomaly detection methods, especially distance-based approaches, do not work well [29]. As pointed out by Pang et al., anomaly detection methods using neural networks can detect anomalies in high-dimensional data well [13]. Therefore, when studying high-dimensional data, we recommend anomaly detection approaches such as autoencoders and deep one-class classification [13, 21].

For the domain experts, the evaluation of records suggested by anomaly detection methods could be simplified by highlighting anomalous variables. In our experiments, domain experts reviewed the entire anomalous record to find implausibilities, resulting in a very time-consuming review process. To reduce the manual effort, explainable anomaly detection methods could visualize the anomalous variables and values of selected records [30]. Furthermore, explanations for the selected records could increase transparency and acceptance of anomaly detection methods by domain experts.

### Limitations

We performed our experiments on only one real-world dataset. Nevertheless, we expect similar results for datasets from other cancer registries, because the size of our dataset was large and the data processed in cancer registries are highly standardized across countries [23].

We did not perform multiple runs of our experiments because of the high manual effort required to review the records by domain experts. Thus, we did not evaluate the properties of different randomly selected samples, the random initialization of the weights and biases of the neural network, and the random selection of the mini-batches to train the neural network.

Different domain experts may evaluate the same record differently. To increase the consistency of the domain experts’ evaluations, the experts reviewed the records according to standardized guidelines. In addition, all experts reviewed records from all samples. Each expert, however, reviewed a different number of records from each sample. Thus, the potential bias introduced by the individual domain experts affected all methods, but to varying degrees.

## Conclusions

We applied two unsupervised anomaly detection methods, FindFPOF and an autoencoder, to a real-world categorical dataset to find implausible electronic health records in cancer registries. Records suggested by the anomaly detection methods were reviewed by domain experts in a real-world scenario.

Both anomaly detection approaches performed well in detecting implausible electronic health records. For samples of 300 records, $28\%$ of the anomalous records are implausible. This corresponds to a precision of $28\%$ for FindFPOF and the autoencoder and is higher than the $8\%$ of implausible records in the random sample. Furthermore, the methods investigated select more colorectal tumor records, since this tumor localization has the highest percentage of implausible records in the random sample. Finally, FindFPOF has a higher precision than the autoencoder when fewer than 100 records are suggested for review by each method.

In conclusion, unsupervised anomaly detection can significantly reduce the manual effort of domain experts to improve data quality. In our experiments, the manual effort was reduced by a factor of approximately 3.5 compared to evaluating a random sample.

## Data Availability

The data supporting the results of this study are available from the Cancer Registry Rhineland-Palatinate, but there are restrictions on the availability of these data, which were used under license for the current study, and are therefore not publicly available. However, the data are available from the authors upon reasonable request and with the permission of the Cancer Registry Rhineland-Palatinate.

## Abbreviations

FPOF: Frequent Pattern Outlier Factor
ICD-10: International Statistical Classification of Diseases and Related Health Problems, 10th Revision
ICD-O: International Classification of Diseases for Oncology
